# Relationship of Reduced Pain Intensity and Improved Quality-of-Life with Menstrual Migraine with Aspirin, Acetaminophen, and Caffeine Combination †

**DOI:** 10.3390/healthcare13091032

**Published:** 2025-04-30

**Authors:** Ashoke Mitra, Mako Araga, Abhay Aher, Jay Xu, Gilbert Shanga, Billy Franks, Richard Petruschke

**Affiliations:** 1 Haleon, Warren, NJ 07059, USA; mako.x.araga@haleon.com (M.A.); abhay.x.aher@haleon.com (A.A.); jay.x.xu@haleon.com (J.X.); gshanga@gmail.com (G.S.); richard.x.petruschke@haleon.com (R.P.); 2 Haleon, 3811 LP Amersfoort, The Netherlands; billy.j.franks@haleon.com

**Keywords:** headache, menstruation, migraine, over-the-counter, pain intensity, quality-of-life

## Abstract

**Objective:** The objective of the current post hoc analysis is to evaluate whether the combination of acetaminophen, aspirin, and caffeine (AAC) is more effective than placebo in relieving the pain intensity for and improving the quality-of-life (QoL) of subjects with menstrual migraine (MM). **Methods:** This analysis evaluated the impact of AAC (n = 85) versus placebo (n = 100) in relieving the pain intensity for and improving the QoL of subjects with MM during baseline and at 0.5, 1, 2, 3, 4, and 6 h post treatment. Subjects reported their pain intensity using a 4-point scale and QoL using a 5-point scale. A lower score indicates reduced pain intensity and improved QoL. **Results:** A statistically significant difference between the AAC and placebo groups (*p* ≤ 0.001) was observed in pain relief after 1 h (40% vs. 14%), 2 h (56.5% vs. 24%), 3 h (63.5% vs. 31%), 4 h (65.9% vs. 34%), and 6 h (64.7% vs. 31%) post treatment. Similarly, a significantly higher proportion of subjects reported improved QoL at 1 h (48.2% vs. 28.0%; *p* ≤ 0.005), 2 h (61.2% vs. 40.0%; *p* ≤ 0.005), 3 h (68.2% vs. 44.0%; *p* ≤ 0.001), 4 h (67.9% vs. 39.0%; *p* ≤ 0.001), and 6 h (64.3% vs. 37.0%; *p* ≤ 0.001) post treatment. The mean pain intensity and QoL scores reduced, while the relative pain intensity and QoL (difference between the AAC and placebo groups) increased with time and was sustained for 6 h. **Conclusions:** The rapid onset and sustained effect of AAC make it a potential option for managing headaches and other symptoms, and to improve the QoL of subjects with MM.

## 1. Introduction

Menstrual migraine (MM) with or without aura can be classified as pure MM or menstrual-related migraine. According to the Appendix of The International Classification of Headache Disorders (third edition), in cases of pure MM, migraine attacks happen exclusively during day 1 ± 2 (days −2 to +3) of menstruation. With menstrual-related migraine, attacks happen on day 1 ± 2 of menstruation, and may also occur on other days of the menstrual cycle [1,2,3]. MM is characterized by recurring episodes of headaches and other associated symptoms such as nausea, vomiting, photophobia, and phonophobia [2].

According to a Global Burden of Disease study from 2019, migraine ranks second as the leading cause of years lived with disability worldwide. The global 1-year prevalence of migraine (year 2022) is around 14–15% [3,4]. Globally, the prevalence of migraine is two-fold more in women than in men [3,5]. A study utilizing data from a National Health Interview Survey from 2015 reported that over a 3-month period, the prevalence of migraine and severe headache among the United States adult population was 20.7% in females and 9.7% in males [6]. Migraine prevalence is even more pronounced after menarche, with three-fold higher rates constituting 70% of all migraine cases worldwide [3]. A recent study by Brown et al. in the United States reported that among women diagnosed with migraine, 6.2 million experienced MM. This corresponds to a prevalence of 31.0% among all women and 52.5% among premenopausal women [7]. More than half of women (56.2%) with MM reported moderate to severe disability associated with their migraines [7].

**Figure 1:**
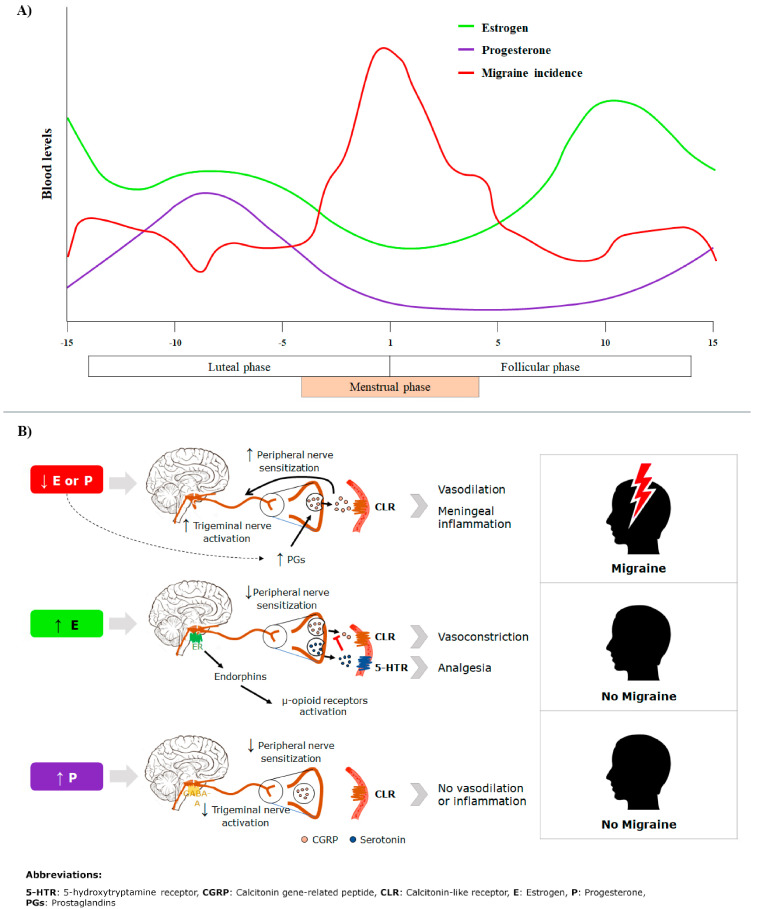
(**A**) Migraine occurrence and blood levels of estrogen and progesterone and (**B**) pathophysiology of menstrual migraine.

The incidence of migraine peaks on or around the first day of menstruation (Figure 1A) [8]. This phase is characterized by a significant decline in estrogen levels. Previous studies have demonstrated an association between the incidence of migraine and a reduction in estrogen levels during the menstrual cycle [9,10]. Figure 1B describes the pathophysiological pathways proposed to be involved in the occurrence of MM [8,11]. Estrogen receptors (ERs) on trigeminal ganglia, which convey migraine pain, may be sensitive to variations in estrogen levels. Significantly higher expressions of ER is reported on the trigeminal ganglia of females compared to males [12]. Estrogen regulates the sensitization of trigeminal neurons by modulating neuropeptides like calcitonin gene-related peptide (CGRP), which cause vasodilation and inflammation upon binding to blood vessels, leading to pain [13,14]. Reduced estrogen levels increase susceptibility to prostaglandins, facilitating neuroinflammation and the release of neuropeptides [11]. Estrogen enhances serotonin expression, which binds to 5-hydroxytryptamine receptor (5-HTR) and prevents CGRP’s pain-promoting effects [15]. Estrogen also modulates the μ-opioid system. Reduced estrogen levels during the late luteal phase reduce the system’s pain-relief capacity [16]. Progesterone also helps to reduce pain by enhancing the gamma-aminobutyric acid (GABA) receptor activity [8,11]. The estrogen withdrawal hypothesis is a widely discussed theory regarding migraine physiology and onset, but it is still not completely understood. Research has shown that perimenstrual estradiol administration can significantly reduce migraine frequency; however, some studies have found no apparent benefit from estradiol treatment. Given that migraines are multifactorial, affected by genetic, neurobiological, environmental, and hormonal factors, future research should aim to uncover the complex interactions between these elements and estrogen to improve the understanding of the condition [17].

Menstrual migraine can be managed via prophylactic or acute symptomatic treatment. A total of 63.9% of MM subjects in the United States reported the use of acute symptomatic treatment [7]. Headaches during migraine can typically be addressed using prescription medications such as triptans or over-the-counter (OTC) products such as nonsteroidal anti-inflammatory drugs (ibuprofen), and combination analgesics such as acetaminophen, aspirin, or caffeine (AAC). Though triptans are very effective for moderate to severe migraines, they may be expensive [8] if not covered by insurance and require a prescription in many countries. Thus, OTC drugs offer the advantages of cost-effectiveness, easy access, and a well-defined safety profile [18,19]. AAC was the first OTC drug approved by the FDA for treating migraine [20]. In a study by Diener et al., 2005, three independent headache episodes were treated for every patient: first, a headache attack with their usual analgesic, followed by two headache episodes with the investigational medication. The results showed that subjects treated with the AAC combination had a slightly but statistically significantly shorter time for 50% pain relief compared to dual therapy (aspirin + acetaminophen), monotherapy (aspirin or acetaminophen or caffeine), or a placebo [19,21]. Similarly, another study demonstrated that AAC provided superior efficacy (measured as weighted sum of pain relief scores at 2 h post treatment) and a faster speed of onset compared with ibuprofen and a placebo in acute migraine [22]. Silberstein et al., 1999 reported that AAC significantly reduced pain intensity, photophobia, phonophobia, and functional disability in subjects with MM [23]. The study reported relief from pain and associated symptoms. However, it did not include data on the impact of AAC or the placebo on the quality-of-life (QoL) of subjects with MM, as it was not part of the analysis plan. Migraine increases the burden on patients’ daily lives [24] and significantly affects their QoL [25]. This post hoc analysis evaluated whether AAC is more effective than a placebo in relieving pain intensity and improving the QoL of subjects with MM [26].

## 2. Methods

### 2.1. Study Population

This post hoc analysis was conducted using the data from Silberstein et al., 1999 [23]. The inclusion criteria were as follows: (1) subjects met criteria for migraine with or without aura based on the International Headache Society’s diagnostic criteria [27], (2) aged ≥18 years, (3) experienced moderate to severe MM pain, were in good overall health, and had a history of migraine headaches occurring at least once every two months but no more than six times per month over the past year, and (4) the intensity of headache was at least moderate when untreated. Subjects with an incapacitating disability (attacks usually required bed rest for more than 50% of the time during an attack) and those who usually experienced vomiting in 20% or more of attacks were excluded [23]. At the time subjects were treated for a migraine attack, they reported their menstrual status. Based on this, migraines were classified as menstruation-associated migraine/MM or migraine not associated with menstruation. The study focused on 967 female subjects and categorized them into having MM (n = 185) or migraine not associated with menstruation (n = 662). Subjects were treated with 2 AAC tablets (acetaminophen 250 mg, aspirin 250 mg, and caffeine 65 mg per tablet) or 2 identical placebo tablets. They were instructed to take the study drug only if the headache reached at least moderate intensity and were asked to avoid taking rescue medication for 2 h, if possible. Subjects documented all symptoms in a diary during the attack, which the investigator later reviewed to confirm that the treated headache was a migraine [23]. This post hoc analysis included a total of 185 women with MM either treated with AAC (n = 85) or the placebo (n = 100).

### 2.2. Outcomes [23]

The primary outcome was the QoL at 1 h post treatment. The secondary outcomes were pain intensity or QoL up to 6 h post treatment. Additionally, the absolute and relative changes in scores at 1, 3, and 6 h post treatment were calculated.

At baseline and at 0.5, 1, 2, 3, 4, and 6 h post treatment, subjects reported their pain intensity using a 4-point scale: 0: none, 1: mild, 2: moderate, and 3: severe. This 4-point scale is validated and significantly correlated with the numerical rating scale (NRS) and the visual analog scale (VAS) [28,29]. Migraine-associated disability is correlated with QoL [30]. The QoL categories used in this analysis were based on the functional disability status of subjects and rated using a 5-point scale: 0: able to perform all activities as usual, 1: usual activities require a little additional effort, 2: usual activities require some additional effort, 3: usual activities require a great deal of additional effort, and 4: unable to perform usual activities. For the outcome analysis we defined a binary approach: subjects (%) achieving a pain intensity score or QoL score of either 0 or 1 at different time points were considered an effective response. A lower score indicates reduced pain intensity and improved QoL. The mean pain score and mean QoL score were calculated for 0.5 h to 6 h post treatment.

### 2.3. Randomization

Subjects in the Silberstein et al., 1999 trial were randomized in a 1:1 ratio using a computer-generated schedule. They received either the double-masked study drug (AAC) or placebo tablets [23].

### 2.4. Statistical Analysis

The comparability analysis regarding potential confounders (age, race, treatment history, family history of migraine, pain severity, and associated symptoms) was performed in the study by Silberstein et al., 1999 [23].

The current data analysis was conducted using SAS version 9.4 on a Windows platform. Specifically, to compare the categorical variables (pain intensity score or QoL score of either 0 or 1) between the AAC and placebo groups, and the Chi-square test was used. *p* ≤ 0.005 was considered statistically significant.

## 3. Results

**Figure 2:**
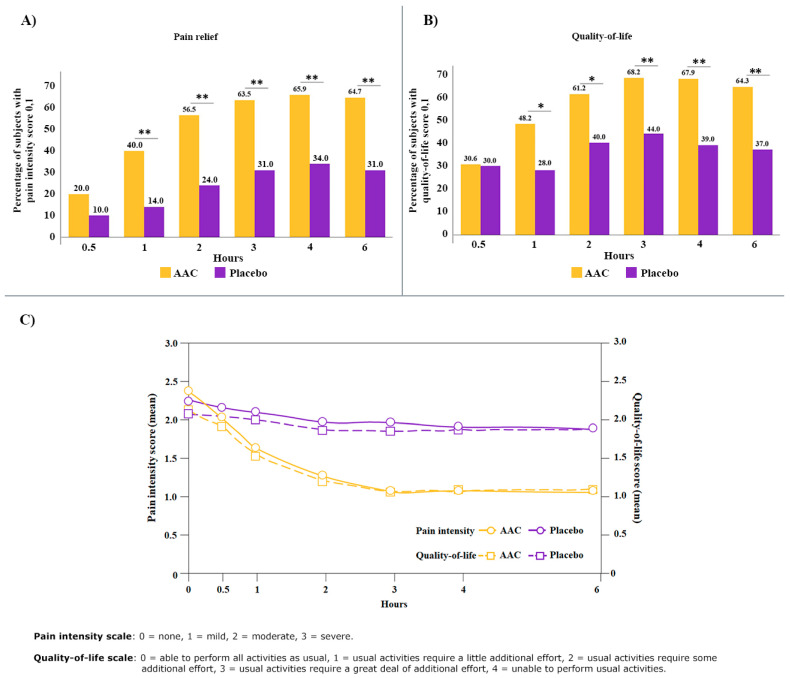
(**A**) Percentage of subjects with pain intensity score of 0 or 1, (**B**) percentage of subjects with QoL score of 0 or 1, and (**C**) mean scores of pain intensity and QoL during 0.5 h to 6 h post treatment in AAC and placebo groups. * *p* ≤ 0.005; ** *p* ≤ 0.001.

The baseline demographics and other characteristics are published elsewhere [23]. There were no statistically significant differences between the AAC and placebo groups with respect to age, race, treatment history, or family history of migraine [23]. The mean age of subjects was 34.0 and 35.4 years in AAC and placebo groups, respectively [23]. At baseline, no significant difference in pain severity was reported between the two groups [23]. A greater proportion of subjects in the AAC group (20%), although not statistically significant, reported a reduction in pain intensity to mild (1) or none (0) within 30 min post treatment, compared to the placebo group (10%) (Figure 2A). A statistically significant difference between the AAC and placebo groups (*p* ≤ 0.001) was observed in the subjects with mild or no pain after 1 h (40% vs. 14%), 2 h (56.5% vs. 24%), 3 h (63.5% vs. 31%), 4 h (65.9% vs. 34%), and 6 h (64.7% vs. 31%) post treatment (Figure 2A). A significantly higher proportion of subjects reported improved QoL (0 or 1 score) in the AAC group at 1, 2, 3, 4, and 6 h post treatment (Figure 2B). The mean pain score at baseline (2.4 vs. 2.3) reduced after 1 h (1.6 vs. 2.1) and 3 h (1.1 vs. 2.0) in the AAC and placebo groups, respectively (Figure 2C). Similarly, the mean QoL score from baseline (2.1 vs. 2.1) improved after 1 h (1.5 vs. 2.0) and 3 h (1.1 vs. 1.9) in the AAC and placebo groups, respectively.

In terms of the relative change between the AAC and placebo groups, there was a 24.6% greater reduction in the pain intensity and a 23.8% greater improvement in the QoL within the first hour (Table 1). By 3 h, the relative pain intensity difference between the AAC and placebo groups further increased to 41.2%, while the QoL score difference increased to 38.1%. Both the pain intensity and QoL values remained consistent throughout the 6 h post treatment.

## 4. Discussion

This post hoc analysis reported the effect of AAC on the pain intensity and QoL in MM subjects. At baseline, the QoL was low, and the subjects required additional efforts to perform their usual day-to-day tasks. Treatment with AAC improved the QoL in a significantly higher proportion of subjects compared to placebo. The burden of hormonally triggered migraines can lead to decreased QoL and significant disability [31]. A recent study by Luo et al. demonstrated that MM patients experience worse health-related quality-of-life (HRQoL) compared to those with non-menstrual migraines [32]. A previous study has reported that the frequency and impact of headaches affect daily lives and have detrimental effects on the QoL of migraine sufferers [33,34]. The current analysis found a correlation between reduced pain intensity and improvement in QoL in subjects with MM treated with AAC, but not with a placebo. The analgesic effect was observed within 30 min and reached significance within 1 h of AAC administration and sustained for up to 6 h, highlighting the rapid and prolonged efficacy of AAC with a single dose in MM patients. A previous study reported that AAC was significantly better than a placebo regarding the weighted sum of pain relief in migraineurs as early as 1 h post treatment [22]. Another study reported a statistically significant difference in pain intensity relative to the baseline after 1 h in the AAC group [21]. The rapid onset of action within 1 h is particularly beneficial for subjects with MM who often experience sudden and severe pain. Furthermore, a recent retrospective cross-sectional study reported that menstrual migraineurs suffered an average of 8.4 headache days per month. Around 42.4% of women included in the study managed the symptoms with non-prescription acute medications [7]. Thus, the ease of obtaining AAC as an OTC offers a convenient and effective treatment option without the need for a prescription. The strength of this analysis is the use of data from three randomized, placebo-controlled, double-blind studies. The limitation includes the post hoc nature of the analysis and unmeasured confounders may have affected the outcome. The study excluded subjects with incapacitating disability (requiring bed rest for more than 50% of the time during an attack) and those experiencing vomiting in 20% or more of attacks, hence the outcome may not be generalized to all subjects. These exclusion criteria were adopted to minimize the probability that the subjects would vomit and not absorb the medication. The use of a 5-point scale for assessing QoL may be considered another limitation of the study, as this scale is not commonly used in current research. The results of this study could be considered hypothetical, as they were obtained from a post hoc analysis. Further research, possibly including prospectively designed studies, is necessary to determine whether the association between analgesia and QoL is maintained over time.

## 5. Conclusions

The AAC combination demonstrated significant efficacy in reducing pain intensity and enhancing QoL for subjects with MM. The rapid onset and sustained effect of AAC make it a potential option for managing headache symptoms in subjects with MM. The availability and reasonable cost of AAC as an OTC medication further enhances its utility, providing patients with a readily accessible, effective, and convenient treatment option. However, these findings may not be applicable to individuals requiring prolonged bed rest or those that experience frequent vomiting during their migraine attacks. Future research should focus on the long-term outcomes of and patient adherence to AAC therapy.

## Figures and Tables

**Table 1:** Change in pain intensity and quality-of-life score at 1 h to 6 h post treatment.

	Baseline	1 h	Absolute Change at 1 h	Relative Change at 1 h (%)	Relative Change (AAC-Placebo) at 1 h (%)	3 h	Absolute Change at 3 h	Relative Change at 3 h (%)	Relative Change (AAC-Placebo) at 3 h (%)	6 h	Absolute Change at 6 h	Relative Change at 6 h (%)	Relative Change (AAC-Placebo) at 6 h (%)
***Pain intensity***													
AAC	2.4	1.6	0.8	33.3%	24.6%	1.1	1.3	54.2%	41.2%	1.1	1.3	54.2%	36.8%
Placebo	2.3	2.1	0.2	8.7%		2.0	0.3	13.0%		1.9	0.4	17.4%	
***Quality-of-life***													
AAC	2.1	1.5	0.6	28.6%	23.8%	1.1	1.0	47.6%	38.1%	1.1	1.0	47.6%	38.1%
Placebo	2.1	2.0	0.1	4.8%		1.9	0.2	9.5%		1.9	0.2	9.5%	

## Data Availability

All relevant data related to this study have been provided in this article.

## Abbreviations

The following abbreviations are used in this manuscript:

5-HTR	5-hydroxytryptamine receptor
AAC	Acetaminophen, aspirin, and caffeine
CGRP	Calcitonin gene-related peptide
ERs	Estrogen receptors
GABA	Gamma-aminobutyric acid
HRQoL	Health-related quality-of-life
MM	Menstrual migraine
OTC	Over-the-counter
QoL	Quality-of-life
